# Cytotoxicity and Epidermal Barrier Function Evaluation of Common Antiseptics for Clinical Use in an Artificial Autologous Skin Model

**DOI:** 10.3390/jcm10040642

**Published:** 2021-02-08

**Authors:** María I. Quiñones-Vico, Ana Fernández-González, Elena Pérez-Castejón, Trinidad Montero-Vílchez, Salvador Arias-Santiago

**Affiliations:** 1 Cell Production and Tissue Engineering Unit, Virgen de las Nieves University Hospital, 18014 Granada, Spain; mariai.quinones@juntadeandalucia.es (M.I.Q.-V.); elenapcastejon@gmail.com (E.P.-C.); salvadorarias@ugr.es (S.A.-S.); 2 Biosanitary Institute of Granada (ibs. GRANADA), 18014 Granada, Spain; tmonterov@correo.ugr.es; 3 Andalusian Network of Design and Translation of Advanced Therapies, 41092 Sevilla, Spain; 4 Dermatology Department, School of Medicine, University of Granada, 18014 Granada, Spain; 5 Dermatology Department, Virgen de las Nieves University Hospital, 18014 Granada, Spain

**Keywords:** antiseptic/antibiotic testing, bioengineered artificial skin substitute, cell viability, epidermal barrier function, regenerative medicine, wound healing

## Abstract

Bioengineered artificial skin substitutes (BASS) are the main treatment used in addition to autografts when skin injuries involve a large body surface area. Antiseptic/antibiotic treatment is necessary to prevent infections in the BASS implant area. This study aims to evaluate the effect of antiseptics and antibiotics on cell viability, structural integrity, and epidermal barrier function in BASS based on hyaluronic acid during a 28 day follow-up period. Keratinocytes (KTs) and dermal fibroblasts (DFs) were isolated from skin samples and used to establish BASS. The following antibiotic/antiseptic treatment was applied every 48 h: colistin (1%), chlorhexidine digluconate (1%), sodium chloride (0.02%), and polyhexanide (0.1%). Cell viability (LIVE/DEAD^®^ assay), structural integrity (histological evaluation), and epidermal barrier function (trans-epidermal water loss, (TEWL), Tewameter^®^) were also evaluated. Cell viability percentage of BASS treated with chlorhexidine digluconate was significantly lower (*p* ≤ 0.001) than the other antiseptics at day 28. Compared to other treatments, chlorhexidine digluconate and polyhexanide significantly affected the epithelium. No significant differences were found regarding epidermal barrier. These results may be useful for treatment protocols after implantation of BASS in patients and evaluating them in clinical practice. BASS represent a suitable model to test in vitro the impact of different treatments of other skin wounds.

## 1. Introduction

The skin is the largest and one of the most complex organs in the human body, representing 15% of total adult body weight [1,2]. Healthy skin is crucial to maintaining physiological homeostasis as it constitutes a protective barrier against external physical, chemical, and biological agents [3].

The epidermis is the outermost layer of the skin, consisting of a renewable epithelium whose main function is to prevent the entrance of foreign substances into the body while allowing water exchange through the skin. The primary cell type in this layer is keratinocytes, which are constantly replaced by basal layer stem cells [2]. The dermis is the thickest layer located below the epidermis. It is a connective tissue formed mainly by extracellular matrix and fibroblasts, which secrete collagen and elastin, providing mechanical strength, flexibility, and elasticity. Beneath this layer the hypodermis is found, an adipose tissue that supplies insulation, cushioning, and an energy storage area [1].

Injuries affecting the skin can disrupt its functions, so it is essential to quickly heal skin wounds [2]. However, there are many situations when the human body cannot correctly heal itself without medical intervention. In case of deep injuries and severe burns, wound healing is slow and incomplete, leaving the body open to infection and poor thermal and fluid regulation, leading to chronic wounds. Currently, the gold standard treatment for these injuries is to use autologous skin grafts to prevent pathogen entry and maintain skin homeostasis. Nevertheless, due to the limit of available native skin, autologous skin grafts can become challenging when these injuries cover a large body surface area [4,5]. That is the reason why skin substitutes were designed to be used in addition to or as a replacement for autologous skin grafts [6]. The most important functions of these substitutes are the prevention of wound infection, reducing pain, promoting wound healing, and the replacement of normal skin to restore its function [3,5,7].

To generate a bioengineered artificial skin substitute (BASS), it is necessary to develop a dermal stroma substitute with human fibroblasts immersed and later, seed the keratinocytes in the upper layer [8,9]. Regarding the BASS dermal stroma, the use of scaffolds is necessary to mimic the extracellular matrix (ECM) in which fibroblasts are immersed. In addition to anchoring cells, the ECM components influence cell survival, proliferation, function [10], rapidly control homeostasis, and modulate the wound environment to maintain an optimal hydration level [11]. Hyaluronic acid (HA) is the most abundant glycosaminoglycan (GAG) in the skin and, along with collagen, one of the most widely used ECM components in wound dressings. HA stimulates cellular proliferation, cellular migration, and angiogenesis, depending on its size [10,12]. Furthermore, it stimulates early inflammation, which is critical for initiating wound healing, but also reduces long-term inflammation. When compared to autografts, BASS based on fibrin-HA showed similar homeostasis parameters and restored epidermal barrier function in a wound mouse model, promoting the formation of a histological architecture very similar to normal skin [13].

Once the skin substitute is ready and grafted into the patient, treatment with antiseptics and antibiotics is crucial to prevent any infection, which is the main risk in these patients [4]. In patients with more than 40% total body surface area (TBSA), 75% of deaths are due to sepsis [14]. The most common microorganisms that cause invasive infections in these burns are *Pseudomonas aeruginosa* and *Acinetobacter baumanii*, followed by *Klebsiella pneumoniae*, *Escherichia coli*, and *Staphylococcus aureus* [15]. Although there is a wide range of protocols [16,17], there is no gold standard antiseptic treatment in burn care.

Antiseptics and antibiotics applied during wound care may affect the viability of skin cells. Few studies have analyzed the impact of antiseptics in cultured fibroblast or keratinocytes [18,19], but no analysis of cytotoxicity and epidermal barrier function have been performed in bioengineered artificial skin substitutes. The main objective of this study was to develop a three-dimensional skin model based on hyaluronic acid scaffold to evaluate in vitro how different treatments (antibiotics and antiseptics), used in clinic, affect cell viability, epithelium integrity, and barrier function. This analysis will provide useful information to select an appropriate antiseptic during the burn care process, but also to treat other skin wounds such as ulcers.

## 2. Materials and Methods

### 2.1. Cell Isolation and Culture

Fibroblasts and keratinocytes were isolated from skin samples (9 cm^2^) from reconstructive surgery donors with the patient’s informed consent in compliance with the requirements for donation of human cells and tissues (Royal Decree-Law 9/2014, of July 4).

Skin samples were transported in Dulbecco’s phosphate-buffered saline (DPBS) from the operating room to the laboratory for the beginning of their processing. In a laminar flow cabin, they were maintained in wash solution for 30 min. Subsequently, scalpel and forceps were used to separate dermis from epidermis. Hypodermis was discarded.

The dermis and epidermis were mechanically processed. The dermis was incubated for 18–24 h in a 2 mg/mL solution of type I collagenase (Gibco, Thermo Fisher Scientific, California, USA) and neutralized with specific medium for dermal fibroblasts culture (DFM). The epidermis incubated with TrypLE Select 10× (Gibco, Thermo Fisher Scientific, California, USA) for 10 cycles of 15 min per cycle and neutralized with specific medium for the keratinocyte culture (KTM) [13]. Cell suspensions were centrifuged at 300× *g* for 10 min. Türk (Sigma Aldrich, St Louis, USA) and trypan blue (Sigma Aldrich, St Louis, USA) solutions were used for counting and cell viability determination, respectively.

Fibroblasts were seeded at a density of 100,000–140,000 cells/cm^2^ (37 °C, 5% CO_2_) at initial processing and at 5000–7000 cells/cm^2^ after passing. The human dermal fibroblasts used as the feeder layer for keratinocytes culture were sub-lethally irradiated (50 Gy). The equipment used was a BIOBEAM 8000 gamma irradiation unit with a source of Cs-137 of 2000 Ci. Dermal irradiated fibroblasts were seeded at 10,000 cells/cm^2^. Keratinocytes were seeded at 20,000–40,000 cells/cm^2^ in the flasks over an irradiated feeder. Cultures were monitored using a Leica DM1000 microscope.

### 2.2. Bioengineered Artificial Skin Substitute (BASS) Manufacturing

**Figure 1:**
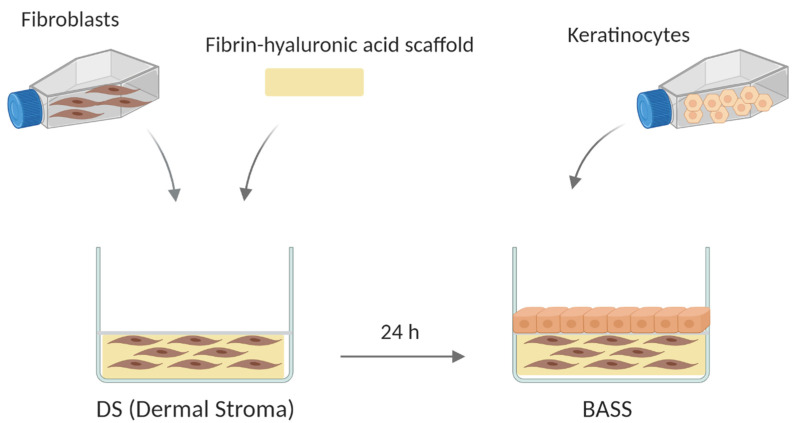
Schematic representation of the bioengineered artificial skin substitutes (BASS) manufacturing process. Created with BioRender.com.

The BASS consists of a two-layer cultured scaffold. The upper layer is an epithelium of keratinocytes and the lower layer is a fibrin-hyaluronic acid matrix containing fibroblasts. The lower layer was developed by mixing 330 µL of human plasma, 62.4 µL of DFM medium containing fibroblasts, 13.4 µL of water (B Braun Medical, Barcelona, Spain), 50 µL of hyaluronic acid (Hyalone, Fidia Pharma, Abano Terme, Italy), 24 µL of calcium chloride (B Braun Medical, Barcelona, Spain, 10 mg/mL), and 20.2 µL of tranexamic acid (Amchafibrin, Rottapham, Spain). The volumes described above correspond to one 500 µL scaffold. After 24 h, keratinocytes were seeded to constitute the upper layer of the BASS and the plate was returned to the incubator for 7 days. Acellular substitutes without cells were fabricated as negative control. Figure 1 summarizes the BASS manufacturing process.

### 2.3. Antiseptic/Antibiotic Test

**Figure 2:**
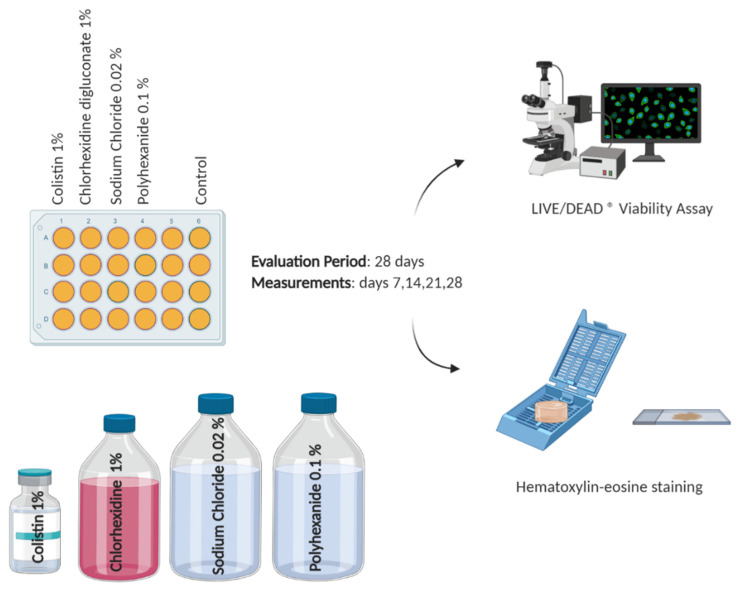
Schematic representation of the antibiotic/antiseptic test. Created with BioRender.com.

A total of 24-well cell culture plates (Thermo Fisher Scientific, CA, USA) of the BASS were treated with three different antiseptics and one antibiotic for 28 days. Controls were left without treatment. Viability assay and histology were performed at days 7, 14, 21, and 28. The antibiotic used was colistin 1% (G.E.S., Genéricos Españoles Laboratorio S.A., Madrid, Spain) and the antiseptics used were chlorhexidine digluconate 1% (HiBiSCRUB^®^, Molnlycke Health Care AB, Madrid, Spain), sodium chloride 0.02% (Sonoma Pharmaceuticals, CA, USA), and polyhexanide 0.1% (B Braun Medical, Barcelona, Spain). Two different protocols of antiseptic application were tested, long (24 h) and short (4 min) application every 48 h, to replicate their clinical use. Therefore, treatment duration was 24 h for colistin and sodium chloride and 4 min for chlorhexidine digluconate and polyhexanide according to previous toxicity studies [19]. The antiseptic/antibiotic test was performed three times (*n* = 3). Figure 2 represents the antibiotic/antiseptic test.

### 2.4. Viability Assay

The primary outcome of this study was to determine cell viability after the different treatments described above. Viability assay at days 7, 14, 21, and 28 was performed using LIVE/DEAD^®^ Cell Viability Assay (Thermo Fisher Scientific, CA, USA). This method allows colorimetric discrimination between living and dead cells. After adding the staining solution, the plate was incubated at room temperature and darkness for 30 min. Then, staining solution was removed, scaffolds were washed with Dulbecco’s phosphate-buffered saline (DPBS, Sigma Aldrich, St. Louis, MO, USA) and placed in slides for fluorescence measurement at 405 nm using a Leica DM2000 microscope. The obtained images were analyzed by the scientific image analysis program Image J.

### 2.5. Histological Analysis

One of the secondary outcomes of this study was to analyze the BASS histological structure. For that, BASS were collected at days 7, 14, 21, and 28, fixed in 4% phosphate-buffered formalin (Merck, Damstadt, Germany), embedded in paraffin, and cut into 4 µm sections. The sections were stained with hematoxylin and eosin (Merck, Damstadt, Germany) to reveal the histological structure.

### 2.6. Epidermal Barrier Function Evaluation

Another secondary outcome of this research was to assess the BASS functionality through the barrier function evaluation. In order to evaluate the barrier function of the BASS, trans-epidermal water loss (TEWL) was measured after 14 days of treatment using a Tewameter^®^ TM 300 (Courage + Khazaka Electronic, Köln, Germany) in 6-well cell culture plates (24 mm diameter, Thermo Fisher Scientific, CA, USA) of the BASS. The Tewameter^®^ probe indirectly measures the gradient of the water evaporation density of the skin through two pairs of sensors (temperature and relative humidity) inside a hollow cylinder. The physical basis for measurement is the law of diffusion discovered by Adolf Fick in 1855: dm/dt = −D*A*dp/dx, where A is the surface (m^2^), m, the water transported (g), t, the time (h), D, the diffusion constant (= 0.0877 g/m(h mmHg)), p is the vapor pressure of the atmosphere (mm Hg), and x is the distance from the surface of the skin to the measurement point (m).

**Figure 3:**
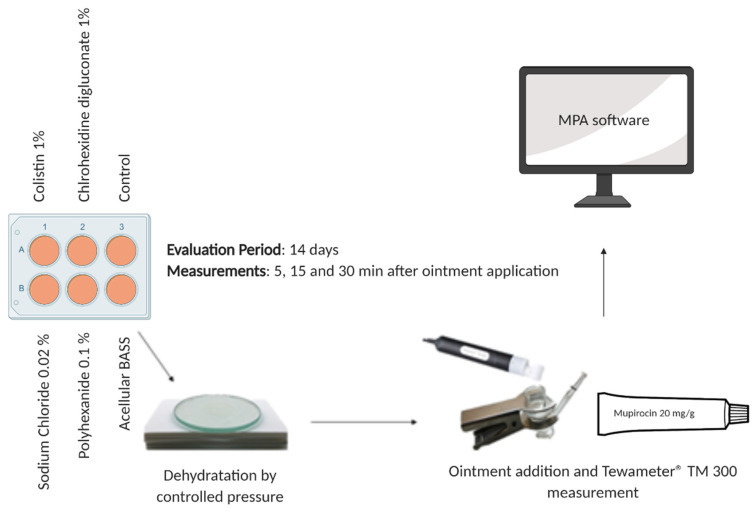
Schematic representation of the trans-epidermal water loss (TEWL) measurement after the antibiotic/antiseptic treatments.

Before taking the measurement, dehydration by controlled pressure of the BASS using a glass disc of 85 g as shot for 2 min was necessary to improve their biomechanical properties [13]. The dehydrated BASS was placed in the Franz cell and 0.5 g of mupirocin 20 mg/g (excipients; macrogol 400 and polyethylenglycol 3350, ISDIN, Barcelona, Spain) was applied. The probe was used to take the measurements (Figure 3) and MPA software (Multi Probe Adapter, Courage + Khazaka Electronic, Köln, Germany) to analyze the resulting data. Mupirocin is a topical antibiotic used to treat superficial skin infections such as impetigo, folliculitis, and furunculosis, as well as other skin diseases, and is widely used during the wound healing process in patients with severe burns or skin ulcers. Figure 3 summarizes the epidermal barrier function evaluation.

### 2.7. Statistical Analysis

The obtained data are presented as the mean ± standard error of the mean (SEM). For the data analysis, the statistical program R was used (R Development Core Team (2011). R: A language and environment for statistical computing. R Foundation for Statistical Computing, Vienna, Austria). A generalized linear model was applied for development of the statistical analysis of the data and normal distribution of the residues was verified. On the linear model, an analysis of variance (ANOVA) factorial was performed, to evaluate the effect of each factor present. Once ANOVA was performed, a post hoc analysis was performed with Tukey’s test for all factors to evaluate the degree of significance when comparing the factor classes. Values of *p* ≤ 0.05 were considered statistically significant.

## 3. Results

### 3.1. Chlorhexidine Digluconate Affects Cell Viability to a Greater Extent Compared to the Other Treatments

**Figure 4:**
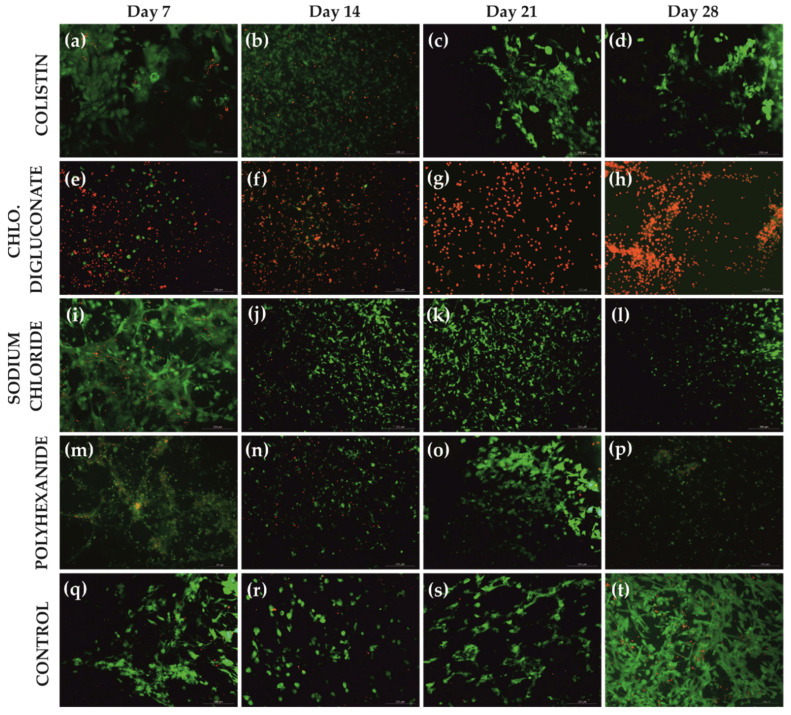
LIVE/DEAD^®^ images of BASS after each treatment and control at days 7, 14, 21, and 28. (**a**–**d**) BASS after colistin (1%) treatment at day 7, 14, 21, and 28 respectively. (**e**–**h**) BASS after chlorhexidine digluconate (1%) treatment at day 7, 14, 21, and 28, respectively. (**i**–**l**) BASS after sodium chloride (0.02%) treatment at day 7, 14, 21, and 28, respectively. (**m–p**) BASS after polyhexanide (0.01%) treatment at days 7, 14, 21 and 28 respectively. (**q**–**t**) Control at days 7, 14, 21, and 28 respectively. Live cells are represented in green and dead cells, in red. *n* = 3. Magnification 10×.

**Figure 5:**
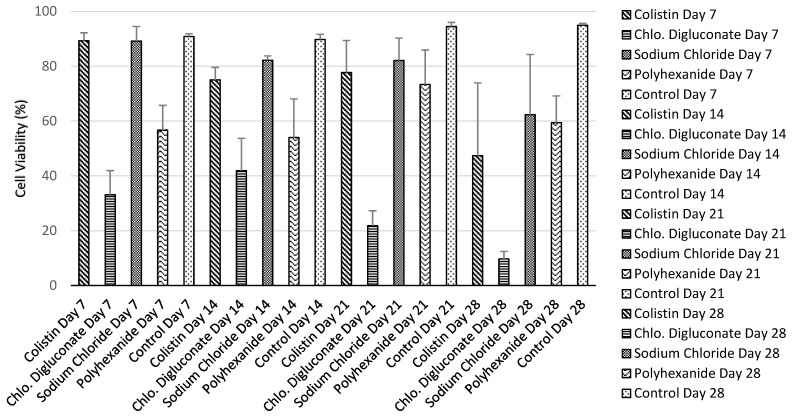
Bar graph of cell viability percentage media for each treatment and control at days 7, 14, 21, and 28. *n* = 3.

Chlorhexidine digluconate greatly reduced cell viability compared to the other treatments. This was reflected in the number of dead cells (Figure 4) as well as in the viability percentage (Table 1, Figure 5).

**Figure 6:**
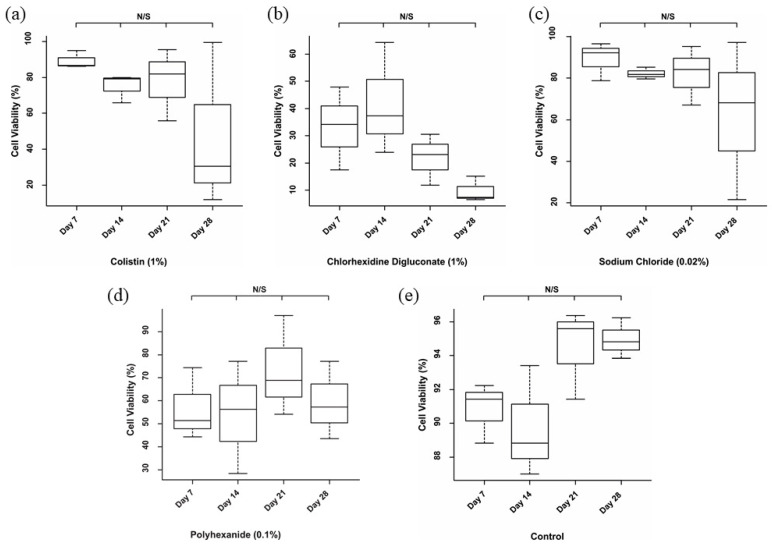
Individual statistical analysis of antiseptic/antibiotic viability test. Boxplot of cell viability percentage after (**a**) colistin (1%) treatment, (**b**) chlorhexidine digluconate (1%) treatment, (**c**) sodium chloride (0.02%) treatment, (**d**) polyhexanide (0.1%) treatment, and (**e**) for the control. *n* = 3. N/S: no significant differences.

**Figure 7:**
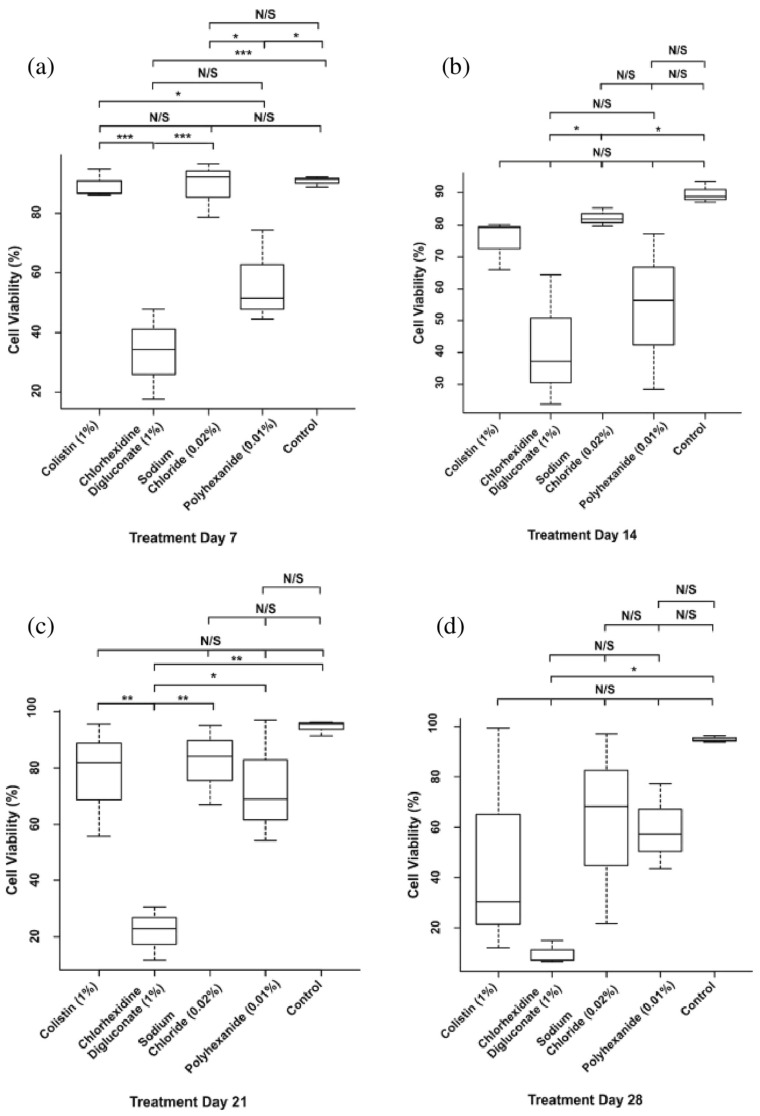
Statistical analysis of the days of treatment in the antiseptic/antibiotic viability (Table 1) treatment. Boxplot of cell viability percentage at (**a**) day 7, (**b**) day 14, (**c**) day 21, and (**d**) day 28 of treatment. N/S: no significant differences; * *p* ≤ 0.05; ** *p* ≤ 0.01; *** *p* ≤ 0.001. *n* = 3. The values of *p* ≤ 0.05 were considered statistically significant.

No significant differences among the days of the evaluation period for each treatment were found (Figure 6). However, the analysis revealed significant differences among the different treatments in each day of the evaluation (Figure 7). Specifically, on day 7 of treatment (Figure 7a), there was a significant reduction in the cell viability after chlorhexidine digluconate compared to colistin (*p* ≤ 0.001) and sodium chloride (*p* ≤ 0.001) treatments, and the control (non-treated BASS (*p* ≤ 0.001); and after polyhexanide compared to colistin (*p* = 0.0272), sodium chloride (*p* = 0.0282) treatments, and the control (*p* = 0.0210). At day 14 (Figure 7b), there was only significant differences between chlorhexidine digluconate treatment compared to sodium chloride treatment (*p* = 0.0472) and the control (*p* = 0.0179). At day 21 (Figure 7c), cell viability was significantly lower after chlorhexidine digluconate treatment compared to colistin (*p* = 00836), sodium chloride (*p* = 0.00497), and polyhexanide (*p* = 0.01415) treatments, and the control (*p* = 0.00127). Lastly, at day 28 of the evaluation period (Figure 7d), there was a significant difference between chlorhexidine digluconate treatment and the control (*p* = 0.0246). Therefore, compared to the control, chlorhexidine digluconate followed by polyhexanide more affected cell viability than the other treatments after the antiseptic/antibiotic test.

### 3.2. Chlorhexidine Digluconate and Polyhexanide Affect the Epithelium Integrity to a Greater Extent Compared to the Other Treatments

**Figure 8:**
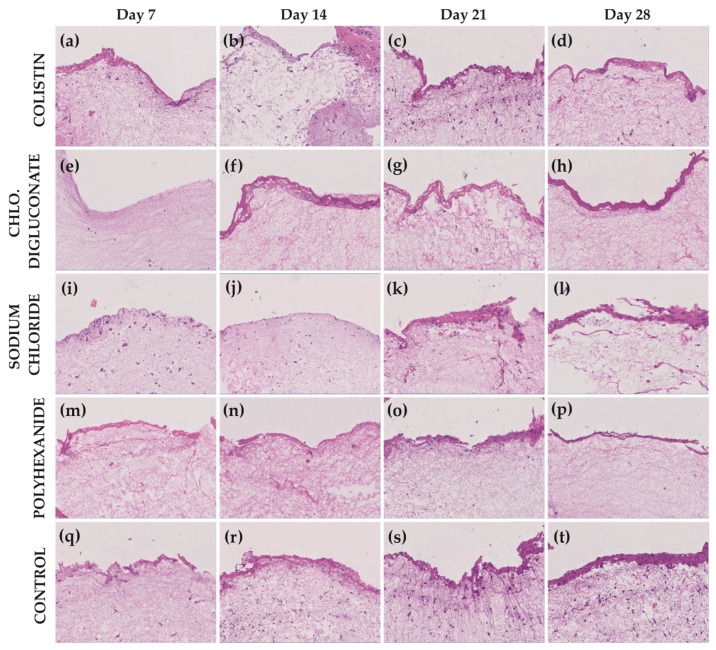
Hematoxylin and eosin staining of BASS after each treatment and control at days 7, 14, 21, and 28. (**a**–**d**) BASS after colistin (1%) treatment at day 7, 14, 21, and 28, respectively; (**e**–**h**) BASS after chlorhexidine digluconate (1%) treatment at day 7, 14, 21, and 28, respectively; (**i**–**l**) BASS after sodium chloride (0.02%) treatment at day 7, 14, 21, and 28, respectively; (**m–p**) BASS after Polyhexanide (0.01%) treatment at days 7, 14, 21, and 28, respectively; (**q**–**t**) Control at days 7, 14, 21 and 28 respectively. *n* = 3. Magnification 10×.

In addition to the viability test, histological analysis was performed to evaluate the skin integrity. As shown in Figure 8, the BASS treated with chlorhexidine digluconate, followed by those treated with polyhexanide, more deteriorated compared to the other treatments, since very few cells were appreciated in the epidermis. Regarding the dermis, an important reduction in fibroblasts population was seen after these antiseptics compared to colistin and sodium chloride treatments. For colistin and sodium chloride, a stratification of the epithelium over time was observed (similar to control group), which suggested the integrity preservation by these treatments.

### 3.3. Skin Barrier Function Was Not Significantly Affected after the Antibiotic/Antiseptic Test

**Figure 9:**
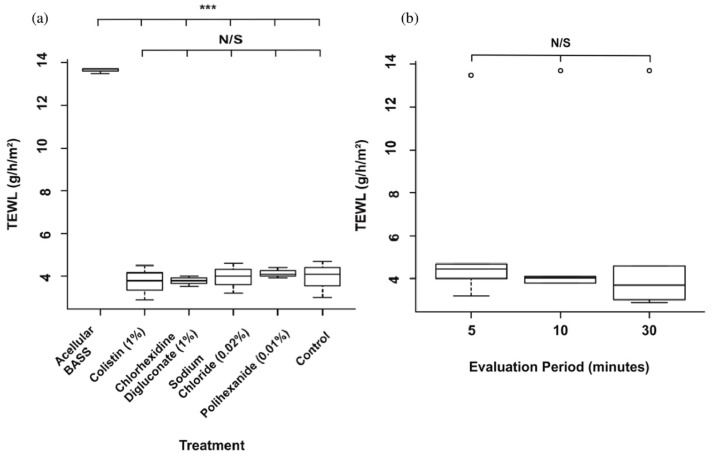
Statistical analysis of TEWL measurement. (**a**) Boxplot of BASS TEWL values regarding the treatment used. (**b**) Boxplot of BASS TEWL values regarding the evaluation period. *** *p* ≤ 0.001, N/S: no significant differences.

As shown in Figure 9, no significant differences were appreciated after mupirocin application between the four groups used and the control (*p* > 0.05) (Figure 9a) and there were no significant differences in TEWL values through the evaluation time (*p* > 0.05) (Figure 9b). However, TEWL values were significantly higher (*p* ≤ 0.001) in the acellular control (absence of barrier) regarding the different treatments and the control.

## 4. Discussion

In this study, chlorhexidine digluconate seriously affected cell viability and epithelium integrity, while no significant differences were observed in the barrier function evaluation.

Bioengineered artificial skin substitutes (BASS) are used in regenerative medicine as an advance therapy for injuries covering large body surface areas, especially burns. These skin substitutes, composed of both a dermis and an epidermis, should be easy to handle, must permanently ensure the skin barrier function, and should not induce a host immune response [1,3,7].

Once the BASS is transplanted to the patient, treatment with antiseptics and antibiotics is crucial to prevent any infection. However, there is no evidence of the impact of these treatments on the viability of the cells that constitute the BASS. If a treatment greatly affects cell viability, this could result in an alteration of skin properties, highlighting the epithelium integrity and its barrier function, which is essential to regulate temperature, water loss and protect from mechanical injuries and external agents [18]. Among all the antiseptics and antibiotics available [16,17], four of the more widely used in clinic were selected to be evaluated in terms of cell viability and barrier function.

Chlorhexidine is a powerful antiseptic with evidence of a beneficial role in burn care [19]. It is a positively charged bisbiguanide at physiologic pH that binds to the negatively charged cell walls of bacteria, leading to a disruption of microbial cell membranes [20]. Several in vitro studies have reported cytotoxicity on cultured cells, whereas in vivo and clinical data results seem to be controversial. In vivo, although it is widely used in clinic due to its broad-spectrum antimicrobial activity, adverse effects have been reported in the literature. For instance, chlorhexidine is occasionally associated with contact dermatitis, and rarely with anaphylaxis and hypersensitivity reactions such as urticaria [21,22,23]. Skin allergic reactions and burns are reported mainly on extremely low birth weight infants, but these adverse effects can occur on adults as well [19,24]. Furthermore, if it contacts the ocular surface, progressive corneal damage can occur, causing toxic effects on the epithelial layers of the cornea and conjunctiva at low concentrations and leading to irreversible keratitis at higher concentrations [24,25]. Middle ear ototoxicity with sensorineural deafness has been reported [24]. In terms of wound healing, the role of chlorhexidine is controversial as well. It has been reported that the use of antiseptics such us chlorhexidine inhibits wound healing in vivo [26], although other reports hold that the concentration of chlorhexidine, which is cytotoxic in vitro, is not cytotoxic in vivo and does not interfere with the re-epithelialization and healing process of the wound [19,27].

Regarding in vitro cytotoxicity, chlorhexidine has significant adverse effects on dermal fibroblast growth [20] and significantly reduces cell migration and survival of myoblasts and osteoblasts [28]. In our BASS model, chlorhexidine digluconate reduced the cell viability to a large extent in comparison with colistin, sodium chloride. and polyhexanide. Compared to the control, chlorhexidine followed by polyhexanide more affected cell viability than the other antiseptics. The reduction in the cell viability resulted in deterioration of the epithelium integrity and this could alter the barrier function. The trans-epidermal water loss is widely used as a marker for skin barrier function in vivo and in vitro [29,30,31]. When skin is damaged, its barrier function is impaired, resulting in a higher water loss [32]. Therefore, if the cell viability of the BASS is reduced and its epithelium is deteriorated, this should be traduced into higher water loss and poor absorption of the dermatological agent used to treat wounds. In our study, no significant differences in TEWL between groups were observed. However, the absence of epithelial barrier in the acellular BASS revealed significantly higher TEWL values compared to the different treatments and the control. Although chlorhexidine significantly reduces cell viability, this was not reflected in the TEWL measurement. More in vivo and in vitro studies with longer follow-up periods may be necessary to assess epidermal barrier function differences between groups and to determine when alterations on epithelial barrier are produced. The use of suitable probes for in vitro long follow-up measurements is highly recommended [30,32,33]. Furthermore, for future studies, the antiseptics and antibiotics treatments could be tested on BASS simulating skin disease models. This could be achieved by damaging the BASS through different methods [34,35] or by developing them using fibroblasts and keratinocytes from patients with different skin pathologies.

To the best of our knowledge, the limitations of this research include the need to improve the barrier function evaluation using suitable probes for in vitro TEWL measurements and to introduce BASS skin disease models that could simulate different types of damaged barriers.

## 5. Conclusions

The role of chlorhexidine in burn care is still controversial since its broad spectrum of action is well-established but effects on wound healing and reepithelization are still contradictory. In this study, chlorhexidine digluconate seriously affected the cell viability and epithelium integrity, while no significant differences were observed in the barrier function evaluation. These results could help determine the appropriate treatment after BASS implantation or antiseptic use for wound healing, although further research is necessary to propose new treatment protocols after implantation and evaluating them in clinical practice. Furthermore, our BASS could be a suitable model to study in vitro the impact of different antiseptics/antibiotics for the treatment of other skin wounds such us ulcers or cutaneous detects.

## Figures and Tables

**Table 1:** Cell viability percentage media for each treatment and control at days 7, 14, 21, and 28; *n* = 3.

Treatment	Day 7	Day 14	Day 21	Day 28
Colistin	89.35%	75.03%	77.7%	47.34%
Chlorhexidine digluconate	33.16%	41.86%	21.81%	9.69%
Sodium chloride	89.15%	82.18%	82.08%	62.34%
Polyhexanide	56.66%	53.99%	73.37%	59.41%
Control	90.83%	89.76%	94.47%	94.96%

## Data Availability

Data is contained within the article.
